# Navigating the Scholarly Literature: A Practical Guide to Searching Effectively (Without Too Much Stress)

**DOI:** 10.1002/cpz1.70138

**Published:** 2025-05-09

**Authors:** Kathryn P. Kohn

**Affiliations:** ^1^ The Claremont Colleges Library Claremont California

**Keywords:** database searching, information sources, research methodology, scholarly literature, search strategies

## Abstract

Effectively searching the scholarly literature is a fundamental academic skill. However, the process can be overwhelming due to the vast amount of available research and the complexity of academic databases. This overview article provides a practical guide to navigating the literature with confidence, outlining key strategies for identifying relevant sources, refining search queries, and troubleshooting common challenges. © 2025 The Author(s). *Current Protocols* published by Wiley Periodicals LLC.

## INTRODUCTION

The scholarly literature is a powerful tool to aid in your research. Whether you are looking for inspiration, developing an understanding of your research context, or trying to solve a problem, there is much to be gained from the work of other scholars in the field. That being said, navigating the literature can be daunting. Where, how, and what should you search? How do you troubleshoot your search when there appear to be too many results or not enough? When have you done all that you can do, and how do you ask for help? In this article, we will navigate these questions and help you build confidence in your ability to effectively search the literature.

## CONSIDERING YOUR TOPIC

So where should we start? While many people, both students and faculty alike, will jump right into searching, it is strongly recommended that you first take a step back to consider your goals and where you are in the research process. Map out your topic and identify the specific information needs that you have, for the project as a whole and in the context of individual searches. Or put another way, what questions can the literature answer for you? By explicitly breaking down your objectives into manageable chunks, you can more easily determine how best to seek out this information, as well as evaluate the success of your search, and if necessary, how best to revise it. Further, having a clear goal in mind will allow you to identify sources best suited to meet those needs and even determine what parts of a source may be most valuable to you at this moment. Ultimately, searching the literature is an iterative process that includes refining your search objectives as well as your approach to navigating the literature.

There are many ways to approach this process, from concept mapping to list making. Regardless of how you go about it, writing down your ideas with a generative/brainstorming mindset can be beyond beneficial. No matter how small or specific your search objective is at the moment, when working in the context of a larger research project, you will need to engage with the literature repeatedly. By documenting your goals, you not only force yourself to engage in the task explicitly but also provide a record that you can rely on in the future. Think of searching the literature as an extension of the work you do in the lab or in the field, and document, document, document. The terms that you put to paper at this moment can help you start to think about the keywords that you will later use to search.

It is not uncommon to start with a broad goal and find that you need to break it down into smaller, more manageable chunks. When searching, you want your goals to be sufficiently defined such that they can help you develop, implement, and troubleshoot your search strategy. This will also allow you to prioritize your needs, and you can start to craft the search itself. What types of sources are you looking for? Where can you find these types of sources? And what approaches to searching will best suit your needs?

That being said, sometimes we have an idea about what we want to learn, but we do not yet have the context of what the literature can offer us. In such cases, consider conducting some exploratory searching where, rather than attempting to find specific information, you are looking for inspiration, identifying research trends and themes, and starting to consider what researchers, organizations, and disciplines are relevant to the topic. For now, just know that we will be discussing this in more detail later.

## TYPES OF SOURCES

Before we go any further, it is necessary to discuss the types of sources that you may find beneficial. As part of the research process, scholars will publish a variety of research outputs that can serve you in various ways. By having a sense of the types of sources available to you, and how they differ from each other, you will be better positioned to seek out those that are best suited to your goals using appropriate search tools and strategies.

### Conference Products

Consider a graduate student conducting a research project. They will likely start by presenting their research at conferences, even while it is still a work in progress. As part of this process, the conference may publish abstracts, conference papers, digitized posters, or recorded presentations. These sources are typically not formally peer‐reviewed, but they can provide us with an early understanding of cutting‐edge research. While these types of information sources can sometimes be found using academic databases, they may also be hosted on conference websites, individual researchers’ pages, or institutional repositories, making web‐based searches a valuable strategy for finding them.

### Original Research Articles

A cornerstone of scholarly publishing is the original research article, which objectively describes an experiment or set of experiments that result in the stated findings and conclusions. They should contextualize the research within the broader scholarly literature, describing any limitations, and discuss implications of the research findings. Some research articles are published alongside supplementary information, while others will refer to specialized repositories that host datasets or detailed experimental protocols. Reaching out to the corresponding author can be another route by which this information is found.

The publication of original research articles relies on the process of peer review, which helps ensure that the research is reliable and clearly described. However, this process can be lengthy, as manuscripts often undergo multiple rounds of review and revision. This means that there may be a significant delay between when research is first discussed at a conference and when the peer‐reviewed paper is published.

### Review Articles

Another extremely valuable source type is a review article. Rather than describing experimentation in depth, the author(s) will synthesize the state of the field on a given topic by providing a high‐level overview of relevant research. Specific types of review articles, referred to as systematic or integrative reviews, establish a replicable search methodology to gather all relevant literature and use quantitative or qualitative analysis techniques on the identified studies in order to answer a specific research question. However, even more informal review articles can support researchers' understanding of the conversations, themes, and gaps in the literature, and provide explicit recommendations for future research. For this reason, seeking out review articles related to your research interests can provide a fabulous starting point, as well as many leads for furthering your literature searching.

### Other Source Types

There are so many other types of sources that can be particularly beneficial depending on your research topic. Further, not all of these are produced within the scholarly publishing landscape. For example, an organizational report or government policy statement may be critical to describe the need of your research. Some disciplines rely strongly on technical standards and experimental protocols. Also, it is not uncommon to engage deeply with resources to further your own understanding of a topic, even if you never end up citing it within a publication. While some of the most common source types used in biology are described in Table 1, you should not limit yourself to these. Just remember to consider that different source types are created in different contexts and for a variety of purposes. You can then use them in ways that keep these factors in mind.

**Table 1:** Types of Sources You May Encounter in Your Research

Source type	Characteristics
Conference products	Describes work at varying stages of completion; examples include presentation abstracts, digitized posters, conference papers, recorded presentations
Original research articles	Researchers objectively describe an experiment or set of experiments
Review articles	Describes the state of the field on a given topic at the time of writing; provides some level of synthesis; may or may not include a formal and transparent methodology for finding, selecting, and analyzing sources
Data sets	Includes dependent and independent variables collected, observed, or calculated; associated documentation that allows for a clear understanding of how the data is defined and where it came from should be provided
Experimental protocols	Detailed description of how to conduct research methods that allow for consistent replication

## WHERE TO SEARCH

Now that you have an idea of what information you are looking for and some of the source types that may be most relevant to your needs, it is time to decide where you will be searching (see Table 2 for an overview of example tools). There are, of course, openly available search tools like Google Scholar that you could start with (see the Internet Resources section for hyperlinks to the tools and websites cited here). However, it is important to consider whether these will be the most efficient or effective choices for your purposes and what other options you may have access to through your library. Most library websites will provide a page dedicated to the databases available, and it can often be filtered based on categories like subject/discipline and format/content type.

**Table 2:** Examples of Search Tools Relevant to Biology

PubMed	USDA National Agriculture Library	Web of Science	ScienceDirect
United States National Library of Medicine (NLM) at the National Institutes of Health	United States Department of Agriculture	Clarivate (an analytics company)	Elsevier (a publishing company)
U.S. government funded search tool; free to access	U.S. government funded search tool; free to access	Subscription based search tool; check with your library (a common alternative is Scopus)	Subscription based search tool; check with your library
Largely biomedical and biochemical literature, life sciences journals, and online books	A wide array of source types, ranging from scholarly articles to reports to books, in areas related to topics such as biotechnology, food and nutrition, aquaculture and fisheries, soil science and more	Curated to include high impact (i.e., highly cited) as well as emerging journals in a wide array of disciplines	Hosts journals and eBooks across four categories: physical sciences and engineering; life sciences; health sciences; and social sciences and humanities
Other tools related to computational molecular biology, health data standards, and human genome research can be found on the broader NLM website	Information previously found in AGRICOLA, PubAg, and National Agricultural Library digital collections can now be found here	Based on your library's subscription, other collections may be searchable on this interface, including BIOSIS Previews	It is unlikely that your library provides full‐text access to every journal and eBook hosted on this platform; remember that services such as interlibrary loan can still facilitate access

When exploring the resources available through your institution, keep in mind that in addition to disciplinary scope and the types of sources that are included, the term “database” can refer to things that work in different ways. For example, citation indexing databases, such as Web of Science and Scopus, allow you to explore a wider range of the literature, because they do not provide access to full‐text sources, instead pointing you to other platforms that provide that service. These databases focus on providing information about sources that will allow you to more easily discover relevant literature and determine if it is right for your project. By comparison, full‐text databases host the source itself. For this reason, they are often tied to a specific publishing company (e.g., ScienceDirect is the platform that hosts journals and eBooks published by Elsevier). This can make access to full text feel more immediate and intuitive, but do not often provide as sophisticated of a search interface or as broad of a scope. That being said, conducting your searches across multiple databases will increase your ability to find a wide range of relevant sources, and the ways in which these databases are built are both valuable and complementary.

While certain academic databases can be cost‐prohibitive for various institutions, you can always rely on access to government supported search tools like PubMed and the USDA National Agriculture Library, even after you graduate. Additionally, these databases may be especially suited to your project if you are interested in exploring government funded research through peer‐reviewed articles or government reports. Keep in mind that while these tools are free to search, they may point you to literature that is not free to access. For this reason, you may need to seek out additional support to understand the best way to access these tools or the full‐text items found using them. Students will almost certainly have access to a librarian who can help them with this process, as do some research institutes and other organizations. Otherwise, you can explore whether your institution has a budget for purchasing access to individual sources or explore research published under an open access license.

## HOW TO SEARCH

### Consider Your Keywords

It is important to remember that the terms you use while searching are going to directly dictate what information you find. While search engines like Google have expensive, proprietary algorithms built into them that use natural language processing that allows you to input full sentences or questions, many databases will (almost) exclusively return sources that include the exact terms you provide. For this reason, it is not only critical to spell things correctly, but to also consider how each term contributes to your current goal. Remove any extraneous terms. For example, if you search *the effect of carbon dioxide on global temperatures*, sources will most likely have to contain all the terms highlighted in green and, depending on the database, the yellow terms as well. Further, while each term will need to be present, the order and placement of these terms can vary widely from source to source. For this reason, words like effect, which are only made meaningful in the context of a phrase or sentence, may not be effective keywords unless you are willing and able to control how they appear in combination with other terms (more on that later). For these reasons, it would be more productive to start our search with a more focused selection of terms (i.e., carbon dioxide global temperatures.)

Another thing that can be surprisingly easy to forget is to be sure to reflect on your immediate research need and make sure your keywords align with that objective. This pertains to both the inclusion and exclusion of terms. Do not force yourself to explore a large set of search results while leaving out a critical component of your need. And while certain terms may be necessary to the project as a whole, they may not be relevant to the search you are currently conducting. In an ideal world, our search results meet our current research need without an overwhelming number of irrelevant results. And while we want our search results set to be focused, we want to avoid missing relevant literature. For this reason, try to think creatively about how various researchers may incorporate alternative terms to what you are used to or gravitate towards. Whether this is due to differences in disciplinary affiliation, target audience, or personal preferences, incorporating synonyms and related terms will only make your searches more comprehensive. Further, there will be times when using more expansive terms in one aspect of your search will allow you to be more specific regarding another component. For example, while looking for transferable information, it may be useful to search for literature studying organisms or locations that share similar characteristics to your research topic in order to identify relevant methodologies suitable for your context.

Overall, thoughtful consideration of the combination and selection of keywords for any given search in alignment with your current objective cannot be understated. For this reason, it is strongly recommended that you maintain a keyword list. This way you can more easily keep track of your search terms across the course of your project, reducing the cognitive effort required to troubleshoot your searches by providing yourself with a toolbox of sorts. Consider using resources that you already have, i.e., anything from course materials to research literature you have already found, to come with disciplinary specific terms. And while you are searching and reading, pay attention to the terms being used and note what keywords have been assigned to individual sources either by the author or the database.

### Constructing Advanced Search Queries

It is also important to consider how your search terms will be combined in order to effectively query the database. An overview of common search operators can be found in Table 3. Let us start with the Boolean operators: AND, OR, and NOT. These specify how your keywords relate to each other and provide a logic to your search. Terms combined with the AND are all required to appear in the results, whereas those combined with the OR operator allow for some variation among the results since only one of the terms will be necessary, although both can appear in a source. For example, in our previous search we could include the molecular formula of carbon dioxide as an option that can be present in place of the written‐out term by searching *(carbon dioxide OR CO2) AND global temperature* (note that I purposefully did not write CO_2_, because this kind of formatting does not generally impact your search). This expands our search leading to more results, but in a way that makes the search more comprehensive and allows us to be specific in other ways. When you start to incorporate the OR operator, do not forget to include parentheses as these help to maintain consistency between your interpretation of your search and the underlying logic of the database. As you may remember from math, *(a OR b) AND c* is not the same as *a OR (b AND c)*.

**Table 3:** Overview of Common Search Operators

Strategy	Description	Example	Things to consider
AND	Connects two terms that must both be present in each returned result	carbon dioxide AND global temperature	In many databases, AND is implicit, which is why we do not have to write carbon AND dioxide AND global AND temperature
OR	Connects two terms for which at least one must be present in each returned result	(carbon dioxide OR CO2)	Placing parentheses around your terms helps to ensure that the database is interpreting the connections between keywords in the same way you are
NOT	The term following NOT cannot be present in the returned result	nursing NOT lactation	Use sparingly so as not to inadvertently exclude relevant literature
Phrase searching (quotation marks)	Connects multiple words to be searched as one term, requiring that they appear in that exact order and form within each returned result	“global temperature”	Some databases will automatically incorporate term variations such as singular/plural or variations in spelling based on American and British English; phrase searching inhibits that
Truncation (depends on the database)	Allows for variability within a term at a specified location	filt* → filter, filters, filtered, filtering, filtration, filtrant, etc.	The very nature of this approach can lead to unexpected things happening; make note of how this manifests in the results

While AND and OR allow for some flexibility in how terms are included within your search results, NOT will exclude sources where a particular term is present. It is important to use this strategy with a degree of caution as terms can appear for many reasons. For example, you may not be studying a particular microbe, but the authors may have included it as a point of comparison. For this reason, only use this strategy as a solution to a known problem where you have seen evidence that this approach will help direct you to more relevant literature.

Two other strategies that can be helpful are truncation and phrase searching. Truncation is a way to incorporate multiple word endings using a wildcard character. Databases can range widely in what truncation symbols they use and to what degree. For example, in Web of Science, the asterisk (*) represents any number of characters, including 0. This means that a search for filt* could return terms like filter, filters, filtered, filtering, filtration, filtrant, and so many more. The nature of this approach is relatively uncontrolled, so keep an eye out for unexpected search terms. Additionally, you may want to consider whether all word forms are equally relevant. If the verb forms of filter are not desirable, you could adjust the location of the truncation symbol to exclude them by searching for filtr*. These are both examples of right‐handed truncation, but internal and left‐handed truncation can both be possible, as well as the use of particular symbols that represent 1 or 0 to 1 characters. For this reason, consider checking out the documentation for the particular database you are using or reaching out to your librarian in order to better familiarize yourself with your options.

Phrase searching is a strategy that you can use to better focus your searches. By enclosing terms in quotation marks, you indicate that they must appear together and in a particular order. While you could technically use this approach for any phrase, it is considerably more productive for disciplinary specific terms that just happen to incorporate multiple words. If you overuse this technique, you could easily miss relevant literature because you are overemphasizing a single approach to describing a concept. For example, while the phrase *sulfated polysaccharide* is certainly a meaningful one, you may find that there are other relevant phrases where the two terms are not in immediate proximity. Further, an abstract that refers to sulfated modification or sulfated derivatives of polysaccharides, could still be relevant without using any version of the phrase. For this reason, it is helpful to think about this technique (and others such as NOT) as tools that can be employed when there is a specific need to better focus your results. Why exclude potentially relevant results when you already have a limited number at your disposal.

### Exploratory Searching

The techniques described above can both broaden and narrow your search results, which can be especially productive when you have a defined information need. However, there are times when you will be navigating the literature in a more exploratory capacity. In these instances, you may find yourself with a larger results set than you could feasibly review and without a sense for how you could augment your search to be more specific. Now is the time to explore the display features of the database, such as filtering and sorting. Sorting allows you to order the results in various ways, such that you can explore the most relevant (according to the database's specific algorithm), the newest or oldest, the most highly cited, etc. Each option offers a different perspective on your results, and experimenting with multiple sorting methods can help you find the most useful information for your research. By comparison, filtering will allow you to refine the results by only including (or in some cases, excluding) sources that meet certain criteria. This can range from citation‐related information (e.g., publication title, year, author, etc.) to descriptive information provided as a feature of the databases (e.g., source type, study population, methodology, etc.). Ultimately, the filtering and sorting options available will vary significantly from database to database, so explore the features available and consider how you can view your results from different perspectives. Having a varied exploration strategy will allow you to better understand the breadth of information available to you within the results, which will allow you to better formulate future searches.

### Controlled Vocabulary

Some databases can go beyond keyword searching by incorporating a controlled vocabulary or thesaurus, which means that for each concept that is determined to be relevant to the scope of the database, a representative term is identified and defined. Current and historical synonyms will be listed as additional entry points to that same concept. And the entry will be placed in the context of the overall framework, with broader and narrower concepts being identified. For example, PubMed uses Medical Subject Headings (MeSH) which is maintained by the United States National Library of Medicine (NLM). If you are searching for *cancer research* in PubMed, you will notice that some of the results returned do not explicitly contain the word cancer. Rather these sources were labeled with the MeSH term “neoplasms” based on their use of another entry term (e.g., malignancies, tumor, benign neoplasm, neoplasia, etc.). As you can imagine, by automatically applying the MeSH framework to your searches, PubMed increases the number of potentially relevant sources in your results list.

Another feature of the MeSH framework is your ability to search it directly, and connect to it easily via the PubMed interface, as each source has a section where the applied MeSH terms are linked. Looking further at the MeSH term “neoplasms,” you can not only find the official definition and a comprehensive list of entry terms, but also explore a variety of subcategories, including cysts, hamartomas, precancerous conditions, and more. These features can help you learn about additional keywords that you may wish to incorporate in databases that do not include a controlled vocabulary, as well as consider ways to further define and scope your research topic. Further, the MeSH framework includes another dimension, referred to as subheadings, which allows you to search for sources that incorporate a particular perspective or approach (e.g., diagnosis, pathology, virology, etc.). Ultimately, not all terms will be represented within a controlled vocabulary and not all databases incorporate one. However, it is worth keeping an eye out and learning how to use these features when available, as they can take your research to the next level.

### Citation Chaining

So far, we have primarily discussed research strategies associated with keyword searching. However, one limitation of this approach is that you will always be limited to the terms you know and those you pick up along the way. A complementary technique is to explore the literature through the citation network. You have likely done this already, learning more about a topic by tracking down the sources that an author referenced within their own writing. It is relatively straightforward to identify and access these papers, as the necessary information for finding them are situated in the bibliography of the work you are reading. However, some databases will make this even more convenient by including a linked list of the references for each paper.

Better yet, some databases are also capable of linking you to sources that have been published more recently, allowing you to see how a source has been used in the work of others. In this way, when you identify a relevant article that was published several years ago, you can see how research has progressed from there. It is important to remember that a citation is not necessarily an indication of agreement or support for the ideas presented within a work but rather a way of conversing with it. A researcher may cite an article that was foundational to their project, provided justification or context for a decision that they made, or offered an alternative theory that they are arguing against. Regardless, you will get a better sense of the conversations occurring among researchers by reading papers that are connected through citations.

## CONCLUDING REMARKS

In some ways, the strategies discussed in this article are only the tip of the iceberg, because the way you approach a search is going to depend not only on your research interests but your current information needs. By having your objective clearly in mind, you can better identify when you find success and if you are struggling, what type of problem you may be encountering. From there, it is time to enter problem solving mode. Is it a matter of expanding or focusing the scope of your search? Do you need to identify a database or type of source that is better suited to your current needs? Is it time to pivot from keyword searching to navigating the citation network or vice versa? Even experienced researchers can run into trouble finding relevant literature, especially if it is an emerging research topic or even just a topic that is new to them. If you have access to a research librarian, reach out to them for personalized recommendations about where to search, relevant search strategies, how to evaluate the success of your searches, and so much more. College and university libraries, as well as some research institutes, have librarians who are dedicated to supporting researchers in this way, and you can connect with them via email or make an appointment to discuss your project. So check out your library website and start to learn about the resources and services available to you.

### AUTHOR CONTRIBUTIONS

**Katie Kohn**: Conceptualization; writing—original draft; writing—review and editing.

### CONFLICT OF INTEREST

The author declares no conflict of interest.

## Data Availability

No new data was created in writing this article.
